# Evaluation of the Healthy Schools Program: Part II. The Role of Technical Assistance

**DOI:** 10.5888/pcd9.110105

**Published:** 2012-03-01

**Authors:** Margaret Beam, Ginny Ehrlich, Jessica Donze Black, Audrey Block, Laura C. Leviton

**Affiliations:** RMC Research Corporation, Portland, Oregon; Alliance for a Healthier Generation, Portland, Oregon; Pew Charitable Trusts, Washington, DC; RMC Research Corporation, Portland, Oregon; The Robert Wood Johnson Foundation

## Abstract

**Introduction:**

Evidence-based technical assistance may be needed to implement recent federal policy to prevent childhood obesity through the schools. The Healthy Schools Program is the largest school-based obesity prevention program in the United States. The objectives of this study were to evaluate the role of the program's training and technical assistance and to explore other contributing factors in changing school policies, practices, and environments.

**Methods:**

We analyzed interim progress of schools recruited during the 2007-2008 and 2008-2009 school years as of December 2010. Schools reported progress through an online inventory of policies, practices, and school environment. We compared baseline inventories to the most recent follow-up and tracked both training attendance and contact with national experts. To identify the factors associated with progress, we performed regression analysis on school level and demographics, number of months between baseline and follow-up, and technical assistance.

**Results:**

The amount of training and technical assistance was significantly associated with school progress, controlling for school level and demographics, number of months between baseline and follow-up, and school status at baseline. Although all types of schools saw progress, schools in the South had the most progress and urban schools had the least progress.

**Conclusion:**

Evidence-based training and technical assistance were associated with school progress in changing policies, practices, and environment to prevent childhood obesity.

## Introduction

Nearly 1 in 3 children and youth in the United States is overweight or obese (1). In 2010, both the White House Task Force on Childhood Obesity (2) and the Healthy, Hunger-Free Kids Act (3) set new policies for the schools to prevent childhood obesity by promoting a healthy diet and physical activity. To implement these policies, evidence-based training and technical assistance (TTA) are needed. TTA is more effective when it aligns with principles of school improvement and uses language that resonates with administrators (4-7); uses an external facilitator to guide schools through a systemic approach (8-11); takes the school and district contexts into account (4,6,7,12); co-constructs changes together with schools by adapting models to local context (13,14); is concrete, tangible, and adaptable to many settings (15-17); and clearly specifies the roles of district and school personnel, breaking down complex systems into component parts (17). In addition, diffusion theory predicts incremental adoption of innovations, implying a series of improvement efforts over time (18).

These components of evidence-based TTA are central tenets of the Healthy Schools Program (HSP), the largest program in the nation devoted specifically to school-based obesity prevention. The objective of this study was to answer 2 questions: 1) how much and what kind of TTA was statistically associated with school progress in changing policy, practices, and environments, and 2) what other factors were associated with progress. A companion article (19) addresses the amount and types of progress that schools made.

## Methods

### Study design

Schools recruited during the 2007-2008 and the 2008-2009 school years submitted baseline and follow-up information concerning policies, practices, and environment. The dependent variable was change between baseline and the most recent inventory that the schools completed. We used 2-step linear regression models to identify factors that contributed to progress. Institutional review board approval was obtained from the RMC Corporation Human Subjects Protections Committee.

### Participants

HSP relationship managers recruited 1,909 schools, including 4 entire urban districts. Recruitment consisted of signing a contract and attending an initial training session. Schools were considered HSP participants if they took part in TTA, submitted an action plan, or provided follow-up information within a year. Of the 1,514 schools participating in or completing TTA, the study sample of 1,295 (86%) submitted both baseline and follow-up measurement, representing 68% of all recruited schools. Study schools had predominantly low-income and African American or Hispanic students. Urban and rural schools predominated, and 48% were in the South (19).

### Training and technical assistance

HSP provides TTA at no cost for 4 years, although 33 schools completed TTA early. School principals designate representatives to undergo TTA and lead school-level implementation. In line with research findings, HSP provides 3 standardized components of TTA and adapts other components to the individual school. Only the standard components are tracked. HSP adapts the timing and intensity of all TTA based on relationship managers' assessments of needs and opportunities, school action plans, schools' voluntary use of resources, and schools' incremental progress. TTA is provided via telephone, webinar, e-mail, and school visits.

The 3 core components that distinguish HSP from other school obesity prevention efforts are a highly structured change process, training sessions with relationship managers, and the opportunity for TTA with national experts. HSP implements a 6-step change process: 1) formation of a school wellness council; 2) completion of an assessment, the HSP Inventory; 3) local prioritization and action planning; 4) technical resource development and brokering; 5) implementation support; and 6) monitoring and evaluation of progress through updates to the HSP Inventory. Relationship managers lead school representatives through these steps in 9 highly structured train-the-trainer sessions over 4 years. There are 3 sessions in year 1 and 2 sessions each in years 2 to 4. Sessions prepare school representatives to implement the 6-step change process and train other school personnel to make changes outlined in the Healthy Schools Framework (www.healthiergeneration.org/schools.aspx?id=3470). Relationship managers encourage schools to tackle new improvements annually. Relationship managers also arrange contact with HSP's 7 national content experts, who tailor TTA to the specific needs of a school. Schools can request this assistance or the relationship managers can recommend it.

Between sessions, relationship managers coach school representatives through the process. HSP also provides resource brokering support: school representatives receive a biweekly electronic newsletter with information about grant opportunities, new wellness resources, school successes, and current research. Schools can also access a resource database containing more than 800 resources and 8 online topical tool kits, both aligned to the framework and vetted by the American Heart Association's Science Advisory Committee.

### Data collection

Schools submitted information online to the HSP Inventory, whose reliability, validity, and data collection procedures we describe in the companion article (19). We dichotomized and summed responses in 8 content areas to create index scores in each content area and a total index score for each school. These were policy and systems, school meals, competitive foods and beverages, health education, physical education, physical activity outside of physical education, before- and after-school programs, school employee wellness, and total score.

Schools updated their inventories at different rates, and the follow-up inventory was the latest information available about progress as of December 2010. Most schools completed their baseline inventory when they started implementing HSP, and the mean (standard deviation [SD]) number of months between baseline and follow-up inventories was 18.6 (8.6). Thus, the months between baseline and follow-up variables approximates the length of schools' participation in the program at the time they last updated their inventory responses.

We downloaded school demographic data from the National Center for Educational Statistics Common Core of Data (20). School level is a categorical variable defined as elementary, middle, high, and other/missing. Percentage of students eligible for free or reduced-price lunch indicates the predominant income level of the students, and is a continuous variable from which we derived 4 categories (0%-25%, 26%-50%, 51%-75%, and 76%-100%). Primary race/ethnicity is a categorical variable that indicates the predominant racial/ethnic enrollment of the school (white, African American, Hispanic, or Asian). Locale is a categorical variable that indicates the type of community in which a school is located (urban, suburban, town, or rural). Geographic region is a categorical variable based on US Census categorization (Northeast, Midwest, South, or West) (21). Complete demographic data were not available for 40 (3%) of the 1,295 study sample schools.

We developed 2 TTA variables. Number of technical assistance sessions is a count measured by participant attendance sheets. Contact with national experts is a binary variable measured by the experts' contact logs with schools (0 = no contact; 1 = 1 or more contacts).

### Data analysis

By using SPSS 18 (SPSS, Inc, Chicago, Illinois), we created linear regression models for the index of total progress and for each of the 8 content areas. The dependent variables were total gain score and content area gain score, computed by subtracting the baseline index scores from the follow-up index scores. We used listwise deletion to handle missing data.

We first assessed which school characteristics were associated with outcomes by conducting a software-controlled stepwise backwards elimination regression. For this task we used the total index scores for parsimony and because they comprised all individual content area scores. We included variables in the model for school level, percentage of students eligible for free- or reduced-price lunch, primary race/ethnicity, locale, and region. They were used as covariates in all subsequent analyses if they were even marginally associated with progress on the total index gain score (*P* < .20).

For each of the 9 index gain scores, we next created 2-step regression models. In step 1 we used the school characteristic covariates and months between baseline and follow up. In step 2 we added the TTA variables to the covariates used in step 1. The final analyses examined the relationships between schools' follow-up index score and the following variables: baseline index score, months between baseline and follow-up, school level (middle school vs other), locale (urban vs other), region (South vs other), number of technical assistance sessions, and contact with a national content expert. We assessed effect sizes by using Cohen's *f^ 2^*, the variance explained by the independent variables.

## Results

Schools recruited in 2007-2008 had attended a mean (SD) of 6.1 (1.9) training sessions, and 20% had consulted a national content expert. Schools recruited in 2008-2009 had attended a mean (SD) of 4.8 (1.4) sessions, and 25% had consulted a content expert. Three demographic variables (middle school level, South region, and urban locale) were associated with progress on the total index (*P* < .20) and were used as covariates in all subsequent analyses (see the companion article [19] for descriptive statistics).

In the regression analysis for the total index gain score, model 1 includes covariates only, and model 2 adds the TTA variables in step 2 to the covariates used in step 1 (Table). Schools with lower baseline scores made significantly greater progress than schools with higher baseline scores. The number of months between baseline and follow-up was significantly and positively associated with progress. Urban schools made less progress than nonurban schools; 3 of the urban school districts in the sample were consistent with this pattern, but outcomes for a fourth district showed much better progress (data not shown). Southern schools made more progress than schools in other regions, even though southern schools also had significantly higher baseline scores. School level did not explain significant outcome variation. The other school variables remained significant when the TTA variables were added in step 2 (model 2 of Table). Both the number of training sessions completed and contact with a national expert contributed significantly to the outcome, controlling for the other variables. Five school variables (level, locale, region, months between baseline and follow-up, and baseline score) accounted for 19% of the variance, considered a moderate effect size. Including the TTA variables yielded an additional 5% to the explained variance in outcome.

We repeated the regression model for individual content areas (data not shown). For 7 of the 8 content areas, both of the TTA variables contributed significantly and uniquely to schools' progress. That is, schools that attended more training sessions and schools that had received assistance from a national content expert were significantly more likely than other schools to make progress in the 7 content areas, controlling for covariates. One exception was the content area before- and after-school programs. Only contact with a national content expert was a unique contributor to schools' progress (*P* < .001), not the number of training sessions.

The covariates' influence also varied by content area. Months between baseline and follow-up was not associated with school employee wellness, but schools elected to change this area much more quickly than others (data not shown). Middle schools made significantly greater progress than other schools in health education and physical education (*P* < .001) and in before- and after-school programs (*P* = .02). Southern schools made significantly greater progress than other schools in all content areas except health education. Being an urban school was significantly associated with less progress in all content areas except school meals, physical activity outside of physical education, and before- and after-school programs.

## Discussion

HSP worked with schools at their own pace, consistent with diffusion theory. Thus, length of participation as measured by months between baseline and follow-up was associated with progress in all but 1 content area. However, TTA was also associated with outcomes, controlling for school characteristics and length of program participation. TTA helped schools improve more quickly and undertake more improvements, a finding consistent with the literature (8-11). Without coaching, improvement teams tend to stop work after 1 improvement project (22).

Schools with lower inventory scores at baseline made more progress than schools with higher baseline scores, which could represent regression to the mean or ceiling effects in schools with a higher baseline score. However, the southern schools started with better policy, practices, and environments, and they still made more progress than other schools. These findings are encouraging in light of the higher prevalence of childhood obesity and overweight in southern states and their low spending per student for schools (23,24).

Although schools in all kinds of communities made progress, the urban schools generally made less progress. Urban districts tend to be larger and more centralized, so the power to make changes may not reside at the school level. The bureaucracies of urban districts are often seen as impeding, not assisting, school improvements (25). Yet 1 urban district made excellent progress.

Training sessions were not associated with changes in before- and after-school programs, but schools may feel this area is beyond their own expertise and, thus, require an expert to help them develop programs or link to community resources. Length of participation was not associated with progress in school employee wellness because schools addressed this area very early in the process. Representatives said it was easy to do and gave them an early "win."

A limitation of the study is that it examined associations, not causal relationships, between TTA and the content areas studied. In addition, these results have limited generalizability.

Schools reported more progress in improving policies, practices, and environments when they received more TTA from HSP. These findings may help to identify the TTA needed to implement new requirements of the 2010 Healthy and Hunger-Free Kids Act (3), which requires minimum standards to promote less calorie-dense offerings in all foods sold in school, as well as required changes in schools to promote both physical activity and healthy eating, emerging from the 2010 White House Task Force on Childhood Obesity (2) and a host of new state-level laws and regulations (26).

## Figures and Tables

**Table.** Regression Analysis of School Progress on School Demographic Characteristics, Months Between Baseline and Follow-Up, and Technical Assistance, Healthy Schools Program, 2007-2010^a^

Analysis	**Regression Equation**					

	**Model 1 (Covariates Only)**			**Model 2 (Covariates and TTA Variables)**		

	**β**	***t***	***P***	**β**	***t***	***P***
**Step 1**						
Baseline Healthy Schools Program Inventory total index score	−0.32	−13.20	<.001	−0.32	−13.69	<.001
Months between baseline and follow-up	0.26	9.16	<.001	0.14	4.04	<.001
School level: middle school	0.90	1.43	.15	0.85	1.41	.16
Locale: urban	−1.61	−2.97	.003	−1.49	−2.83	.005
Region: South	2.70	4.96	<.001	3.12	5.91	<.001
**Step 2**						
No. of training sessions	NA	NA	NA	0.79	4.49	<.001
Contacts with national experts^b^	NA	NA	NA	4.00	6.92	<.001
*R^2^*	NA	0.20	NA	NA	0.25	NA
Adjusted *R^2^*	NA	0.19	NA	NA	0.24	NA
	*F*_4,1100_ = 53.83		< .001	*F*_6,1100_ = 51.56		<.001

Abbreviation: TTA, training and technical assistance; NA, not applicable.

a n = 1,100 schools. Excludes schools with missing data.

b A binary variable was used, measured by the experts' contact logs with schools: 0 = no contact; 1 = 1 or more contacts.
